# Multi-Element Exposure and Health Risks of Grains from Ambagarh Chowki, Chhattisgarh, India

**DOI:** 10.3390/toxics13010056

**Published:** 2025-01-14

**Authors:** Bhagyashri Wakhle, Saroj Sharma, Khageshwar Singh Patel, Piyush Kant Pandey, Antonela Blažević, Željka Fiket, Sema Yurdakul, Simge Varol, Pablo Martín-Ramos, Hanan M. Al-Yousef, Ramzi A. Mothana

**Affiliations:** 1 Department of Chemistry, Government Nagarjuna Post Graduate College of Science, G. E. Road, Raipur CG 492010, India; wakhlebhagyashri@gmail.com (B.W.); ssharmagr8@gmail.com (S.S.); 2 Department of Applied Sciences, Amity University, Baloda-Bazar Road, Raipur CG 493225, India; pkpandey@rpr.amity.edu; 3 Division for Marine and Environmental Research, Ruđer Bošković Institute, Bijenička Cesta 54, 10000 Zagreb, Croatia; ablazev@irb.hr (A.B.); zeljka.fiket@irb.hr (Ž.F.); 4 Environmental Engineering Department, Suleyman Demirel University, 32260 Isparta, Turkey; semayurdakul@sdu.edu.tr; 5 Geological Engineering Department, Faculty of Engineering and Natural Sciences, Suleyman Demirel University, 32260 Isparta, Turkey; simgevarol@sdu.edu.tr; 6 ETSIIAA, Universidad de Valladolid, Avenida de Madrid 44, 34004 Palencia, Spain; pmr@uva.es; 7 Department of Pharmacognosy, College of Pharmacy, King Saud University, Riyadh 11451, Saudi Arabia; halyousef@ksu.edu.sa (H.M.A.-Y.); rmothana@ksu.edu.sa (R.A.M.)

**Keywords:** grains, health hazards, mineral, potentiality, toxicity

## Abstract

Rice, wheat, and maize grains are staple foods, widely consumed for their mineral and nutritional values. However, they can accumulate toxic elements from contaminated soils, posing health risks. This study investigates the bioaccumulation patterns of 52 elements (including nutrients, heavy metals, and rare earth elements) in various parts (grain, husk, straw, and root) of cereals grown in a heavily polluted region. The results revealed that rice grains exhibited a higher accumulation (Σ33.4 mg/kg) of toxic elements (As, Cu, Cr, Ni, and Pb) than wheat (Σ26.6 mg/kg) and maize (Σ16.2 mg/kg) grains, with the high-yield RI64 cultivar (Σ47.0 mg/kg) being the most susceptible. Across the rice plant, accumulation increased in the order of grain < husk < straw < root. Elements like P, K, Cu, and Zn showed the highest enrichment. Worryingly, the most toxic elements, such as As, Pb, and Cd, exceeded permissible limits across grains, straws, and husks. Health risk assessment indicated that wheat and maize pose greater non-cancer and cancer risks than rice. Despite being grown in a highly contaminated region, the study identifies some rice cultivars like *Luchai* and *Sarna* as relatively safer options due to a lower accumulation of toxic elements.

## 1. Introduction

Cereals are among the most widely produced agricultural products worldwide. Cereals serve as a major energy source for humans, meeting the body’s carbohydrate requirements [1]. Grains (seeds) of *Poaceae* grasses, such as rice, wheat, and maize, are extensively cultivated due to their use as food, nutrient, fiber, minerals, protein, vitamin, and antioxidant sources, and as renewable energy resources [2,3,4,5]. They are the main source of several trace elements (Mn, Fe, Co, Cu, Zn, and Se) needed for the proper growth and development of living organisms [6]. Iron plays a crucial role in oxygen transport, and its deficiency can lead to anemia [7,8]. Cobalt, Cu, and Mo are required for various enzymatic functions [9,10]. Zinc and Se are abundant trace elements in the regulatory, controlling, and catalytic components of numerous proteins [11,12]. In addition, rare earth elements (REEs) are used as feed additives for crop productivity and improving livestock yield [8].

Rice flour is also utilized in the production of various other products, including infant formulas and noodles [13]. India ranks second in paddy straw production, which is predominantly used as fodder [14]. Rice husks, which constitute approximately 20% of the grain’s weight, are a significant source of silica and renewable energy. Additionally, these husks serve as effective adsorbents for the remediation of heavy metals and dyes from wastewater [15,16]. However, these grains, along with straw and husk, can be contaminated with toxic, heavy, radioactive, and rare earth elements [17,18,19,20,21,22,23,24]. Plants grown on metal-contaminated soil can lead to nutritional deficiencies in the populations of developing countries that are already facing malnutrition problems [25]. In addition, heavy metal-contaminated foods cause non-carcinogenic and carcinogenic health issues. The concentration of rare earth and platinum group elements in the environment is increasing due to their wide industrial application but their excess accumulation in plant food may cause cytogenetic anomalies and diseases by increasing ROS production and DNA as well as cell damage [19,20,26].

This study aims to evaluate the potential health impacts associated with cereals grown in the Ambagarh Chowki district (Chhattisgarh, India), selected as the study site due to the contamination of its agricultural soils with heavy metals and rare earth elements from both natural and anthropogenic sources [27,28,29]. To do so, the distribution, accumulation, correlation, and sources of 52 elements (namely Al, As, Ba, Be, Bi, Ca, Cd, Co, Cr, Cu, Fe, Ga, Ge, K, Li, Mg, Mn, Mo, Na, Nb, Ni, P, Pb, Rb, Sb, Sc, Se, Sn, Sr, Te, Ti, Th, Tl, U, V, W, Y, Zn, La, Ce, Pr, Nd, Sm, Eu, Gd, Tb, Dy, Ho, Er, Tm, Yb, and Lu) in field soil and cereals (rice, wheat, and maize grain, husk, straw, and root) were investigated; contamination transfer and translocation factors, toxicity, and health risks were evaluated, along with source apportionment.

## 2. Materials and Methods

### 2.1. Study Area

The soil in the Ambagarh Chowki area (20.78209° N, 80.74117° E), Chhattisgarh, India, originated from the weathering of rocks containing clay minerals such as biotite, chlorite, illite, kaolinite, and goethite [27]. The climate is tropical with high temperatures, reaching a maximum of approximately 47 °C in May. The annual rainfall is ≈100 cm, occurring during the summer months from June to October. The primary crops cultivated in the study area are rice, followed by wheat and other cereal grains. The water and soil in the study area are severely contaminated with multiple elements. Water samples show elevated levels of fluoride, 3.7–27.0 mg/L, and arsenic, 148–985 μg/L [28]. Soil analyses reveal high concentrations of arsenic (9–390 mg/kg), nickel (12–110 mg/kg), copper (35–1571 mg/kg), and lead (13–545 mg/kg) [29]. These high contaminant levels are associated with various health issues in the local population, including fluorosis, melanosis, keratosis, and skin cancer [28,29].

### 2.2. Sample Collection

**Figure 1:**
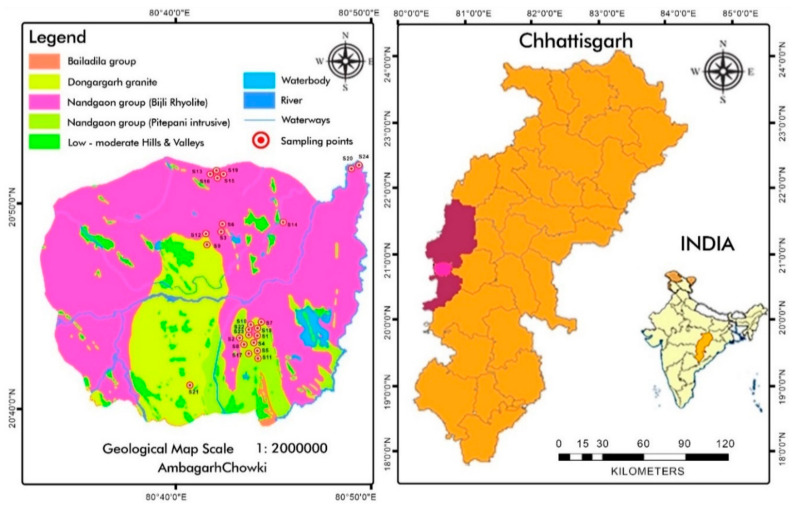
Geographical representation of sampling locations in Ambagarh Chowki, CG, India.

Cereal samples (rice, wheat, and maize) were collected from 24 different locations within the study area (Figure 1) in the summer of 2021. Simultaneously, field soil composite samples from 0−10 cm depth were collected in triplicate from six locations, S_7_, S_8_, S_9_, S_22_, S_23,_ and S_24_ [30], to determine the elemental composition and indices.

The grain, husk, straw, and root components were separated manually. The straw and root samples were washed three times with ultrapure deionized water. The rice husk was removed manually. All samples were sun-dried for one week. The dried samples were crushed into a powdered form by sieving particles of ≤0.1 mm in size. The samples were stored in colored glass bottles, and further dried in a hot oven overnight at 50 °C, then refrigerated at −4 °C until analysis.

### 2.3. Materials

Analytical-grade nitric acid (HNO_3_), hydrochloric acid (HCl), and hydrofluoric acid (HF) were purchased from Kemika (Zagreb, Croatia). Boric acid (H_3_BO_3_) was obtained from Fluka (Steinheim, Germany). The multielement standards containing Al, As, Ba, Be, Bi, Cd, Co, Cr, Cs, Cu, Fe, K, Li, Mg, Mn, Mo, Na, Ni, Pb, Se, Sr, Ti, Tl, V, and Zn (100 ± 0.2 mg/L); Ce, La, Nd, and Pm (100 ± 0.2 mg/L); and Dy, Er, Eu, Gd, Ho, Lu, Sc, Sm, Tb, Tm, Y, and Yb (20 ± 0,4 mg/L) were purchased from Analytika (Prague, Czech Republic), Merck KGaA (Darmstadt, Germany), and Sigma-Aldrich (Milwaukee, WI, USA), respectively. A standard solution of 1000 ± 2 mg/L for Ca and P, and a solution of 1.000 ± 0.002 mg/L (Analytika) for other elements —Ga, Gd, Ge, Nb, Sb, Sn, Te, U, and W— were also incorporated.

### 2.4. Analysis

An Agilent (Santa Clara, CA, USA) 8900 triple quadrupole inductively coupled plasma-mass spectrometer (ICP-MS/MS) available at the Ruđer Bošković Institute (Zagreb, Croatia) was used for the quantification of the 52 elements. It was operated at 1550 W with plasma, auxiliary, and sample flow rates of 15.0, 0.90, and 1.01 L/min argon gas, respectively (Table S1).

The soil subsamples (0.05 g) were digested with a mixed acid solution (4.0 mL HNO_3_, 1.0 mL HCl, and 1.0 mL HF), followed by the addition of 6.0 mL H_3_BO_3_. The extract was further diluted tenfold with 2.0% (*v*/*v*) HNO_3_, and an internal standard (In, 1.0 μg/L) was added.

The plant subsamples (0.07 g) underwent single-step digestion using a mixture of the above-mentioned acids (6.0 mL HNO_3_, and 0.1 mL HF), with the addition of the internal standard (In, 1.0 μg/L).

Mass calibration of the instrument was performed using a multielement solution (Merck KGaA, Darmstadt, Germany) containing the following elements: B, Ba, Co, Fe, Ga, In, K, Li, Lu, In, Rh, Sc, Tl, U, and Y. Calibration curves were generated by external standardization with a series of standard solutions, including a blank sample. Separate standard solutions were prepared for the quantification of selected elements as follows. The standard solutions for trace element determination were prepared by appropriate dilution of a multi-element reference solution (100 ± 0.2 mg/L, Analytika, Czech Republic) containing Al, As, Ba, Be, Bi, Cd, Co, Cr, Cu, Fe, Ga, Ge, Li, Mn, Mo, Ni, Nb, Pb, Rb, Sc, Sr, Te, Th, Tl, V, W, and Zn, in which single element standard solutions of U (1.000 ± 0.002 g/L, Aldrich, Milwaukee, WI, USA), Sb (1.000 ± 0.002 g/L, Analytika, Czech Republic), and Sn (1.000 ± 0.002 g/L, Analytika, Czech Republic) were added. For REEs determination, a multielement reference standard (Analytika, Prague, Czech Republic) containing Ce, La, Nd, and Pm (100 ± 0.2 mg/L) and Dy, Er, Eu, Gd, Ho, Lu, Sc, Sm, Tb, Tm, Y, and Yb (20 ± 0.4 mg/L) was used. For the determination of major elements, standard solution was prepared from single element standard solutions of 1000 ± 2 mg/L (Analytika, Czech Republic) of Ca, K, Mg, and Na, while for P and S, single standards were prepared from reference solutions (Analytika, Czech Republic) containing 1000 ± 2 mg/L of these elements.

### 2.5. Indices

The sodium adsorption ratio (SAR), magnesium hazard (MZ), transfer factor (Tf), and translocation factor (Tr) of elements, average total dose (ATD), chronic daily intake (CDI), cancer risk (CR), and hazard quotient (HQ) were computed as described in the literature [31,32,33,34].

(1) $$\mathrm{SAR}=[Na^{+}]/\sqrt{\{[Mg^{2+}]+[Ca^{2+}]\}/2}$$

where ions are expressed in meq/L.

(2) $$\mathrm{MH}=\frac{\left[Mg^{2+}\right]}{\left([Mg^{2+}]+[Ca^{2+}]\right)}\times 100$$

where ions are expressed in meq/L.

T_f_ = [M_plant_]/[M_soil_] (3)

T_r_ = [M_grain_]/[M_root_] (4)

ATD = Asg × IR(5)

CDI = C_m_ × DI/BW (6)

HQ = CDI/RfD(7)

HI = ΣHQ_i_ (8)

CR_lim_ = RfD × BW/C_m_ (9)

Cancer risk = CDI × CSF(10)

where M_soil_, M_plant_, M_grain_, and M_root_ denote the analyte concentration in soil, plant part (grain, husk, straw, or root), grain, and root, respectively. The variables ATD, Asg, IR, C_m_, DI, BW, RfD, CDI, HI, HQ_i_, CR_lim_, and CSF represent the average total dose, arsenic contamination of grain (in mg/kg), grain ingestion rate (kg/day), mean concentration of the toxic element in food, amount of food consumed per day (0.5 kg/day), mean body weight of an individual (60 kg), reference dose (0.5 kg/day), chronic daily intake, hazard index, summation of HQ of non-carcinogens, maximum allowable food consumption rate (in kg/day), and cancer slope factor (in mg/kg/day), respectively. The Rfd values for Cr, Ni, As, Cd, and Pb are 0.003, 0.02, 0.0003, 0.001, and 0.0035 mg/kg/day, whereas the CSF values of Cr, Ni, As, Pb, and Cd are 0.5, 1.7, 1.5, 0.38, and 0.01 mg/kg/day [33,34].

### 2.6. Quality Assurance/Quality Control (QA/QC) Analysis

Standard reference materials for soil and plant samples (NCS DC 77302 and NCS ZC73018), supplied by the China National Analysis Center for Iron and Steel (Beijing, China), were used for quality control. The ICP-MS detection limits (DL) for Al, As, Ba, Be, Bi, Ca, Cd, Co, Cr, Cu, Fe, Ga, Ge, K, Li, Mg, Mn, Mo, Na, Nb, Ni, P, Pb, Rb, Sb, Sc, Se, Sn, Sr, Te, Th, Ti, Tl, U, V, W, Y, Zn, La, Ce, Pr, Nd, Sm, Eu, Gd, Tb, Dy, Ho, Er, Tm, Yb, and Lu were 6, 0.003, 0.3, 0.002, 0.002, 25, 0.002, 0.002, 0.03, 0.03, 5, 0.002, 0.002, 15, 0.005, 6, 0.25, 0.005, 6, 0.002, 0.03, 50, 0.03, 0.03, 0.002, 0.002, 0.02, 0.003, 0.5, 0.002, 0.01, 0.5, 0.002, 0.001, 0.002, 0.01, 0.002, 1.5, 0.002, 0.002, 0.002, 0.002, 0.002, 0.002, 0.002, 0.002, 0.002, 0.002, 0.002, 0.002, 0.002, and 0.002 mg/kg, respectively.

### 2.7. Statistical Analysis

Correlation analysis was used to measure the strength of the linear relationships between element contents. Principal component analysis was performed for source apportionment, and three principal components with eigenvalues greater than 1 were extracted using Varimax rotation with Kaiser normalization. Statistical analyses were performed using IBM SPSS Statistics version 20 software (IBM Corporation, Armonk, NY, USA) [35].

## 3. Results

### 3.1. Soil Characteristics

The soil in the study area was alkaline (pH 7.5 ± 0.1) and sodic (sodium adsorption ratio, SAR = 5.9 ± 0.9), with a high magnesium hazard (MH), ranging from 43.6 to 60.5%, with a mean value of 53.1 ± 5.9%. The sum of the total elemental content of the 52 elements (*n* = 6) varied from 109,456 to 146,749 mg/kg, with a mean value of 128,595 ± 12,703 mg/kg. In the surface soil, iron content was the highest (37.7%), and crustal elements (Fe, Al, K, Ca, Mg, Ti, Na, and Mn) contributed 98.7% of the total content. The average values of the elements were recorded in the following decreasing sequence: Fe (48,427 ± 9078) > Al (43,083 ± 5211) > K (11,055 ± 1450) > Ca (8643 ± 2299) > Mg (5913 ± 1870) > Ti (5804 ± 682) > Na (2893 ± 604) > Mn (1050 ± 274) > Ba (512 ± 38) > Cr (126 ± 95) > P (124 ± 64) > V (123 ± 19) > Zn (99 ± 16) > Ce (94 ± 10) > Rb (78 ± 8) > Sr (72 ± 12) > Ni (70 ± 21) > Cu (53.0 ± 10.8) > As (48.5 ± 13.7) > Sc (42.7 ± 2.4) > La (42.4 ± 3.4) > Nd (37.6 ± 2.8) > Co (31.5 ± 6.7) > Pb (28.7 ± 3.2) > Y (26.0 ± 2.6) > Ga (17.7 ± 2.2) > Th (15.7 ± 1.4) > Nb (15.6 ± 1.2) > Li (13.7 ± 1.6) > Pr (10.2 ± 0.7) > Sm (7.6 ± 0.6) > Dy (5.5 ± 0.5) > Gd (5.14 ± 0.42) > Er (3.43 ± ) > Yb (3.20 ± 0.37) > U (3.15 ± 0.43) > Ge (3.08 ± 0.40) > Sn (3.01 ± 1.02) > W (2.81 ± 0.70) > Eu (1.60 ± 0.15) > Ho (1.15 ± 0.11) ≈ Tb (0.96 ± 0.08) ≈ Be (0.96 ± 0.08) > Mo (0.92 ± 0.24) > Sb (0.79 ± 0.02) > Tl (0.58 ± 0.05) > Tm (0.49 ± 0.06) > Lu (0.47 ± 0.05) > Bi (0.26 ± 0.04) > Se (0.24 ± 0.10) > Cd (0.21 ± 0.12) > Te (0.05 ± 0.02 mg/kg).

Eighteen elements, including Cd, Ga, Mo, V, Y, and REEs (except Ce), were well correlated (0.67–1.0), probably due to their geogenic origin. However, good correlations (0.71–0.99) of specific groups of elements: As, Pb, Sn, and U; Cr, Li, Mo, and Ni; and Cu, Fe, Ga, Ge, Mo, P, Se, Ti, V, Zn, Eu, Dy, and Er were also observed.

The concentrations of arsenic and other metals in the field soil were found to be higher than those reported in other locations [36,37,38].

### 3.2. Distribution of Elements in Plants

The uptake of elements from root to grain depends on numerous physiological and environmental factors [39]. The distribution of the 52 elements in rice grain (RG), wheat grain (WG), maize grain (ZG), rice husk (RH), rice straw (RS), wheat straw (WS), rice root (Rr), and wheat root (Wr) is shown in Table 1. The concentration range of these 52 elements in RG, WG, MG, RH, RS, WS, Rr, and Wr varied from 10,249 to 58,413 mg/kg, recording maximum content in Rr.

**Figure 2:**
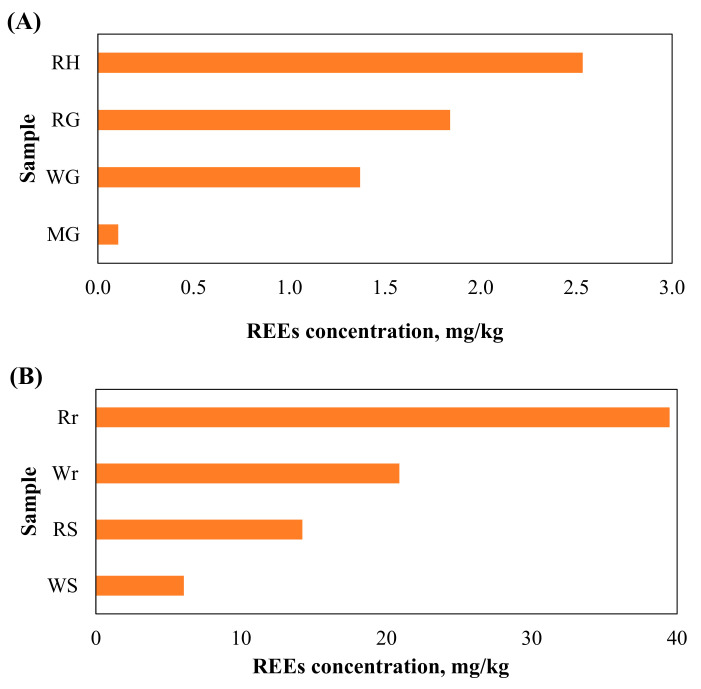
Graphical representation of REEs concentration in rice, maize, and wheat plant parts: (**A**) rice husk (RH), rice grain (RG), wheat grain (WG), and maize grain (ZG); (**B**) rice root (Rr), wheat root (Wr), rice straw (RS), and wheat straw (WS).

The concentration of rare earth elements (ΣREEs) in the plants ranged from 0.11 to 39.5 mg/kg (Figure 2A,B), occurring in the following increasing order: maize grain < wheat grain < rice grain < rice husk < wheat straw < rice straw < wheat root < rice root. The ratio of light to heavy REEs ([ΣLREEs] (lower rare earth elements)/[ΣHREEs] (higher rare earth elements)) ranged from 8.6 to 10.5, with the lowest value in the rice root (Table 1). The individual REEs occurred in the following decreasing sequence: Lu < Tm < Tb < Ho < Eu < Yb < Er < Gd < Dy < Sm < Pr < Nd < La < Ce.

It is worth noting that higher aluminum content was accumulated in rice compared to wheat and maize (Table 1), exceeding the provisional tolerable weekly intake (PTWI) value of 7.0 mg/kg body weight [40]. Elements such as As, Ba, Be, Cd, Co, Cr, Cu, Li, Mn, Ni, Pb, Sb, Sr, Te, Th, Tl, U, and V are considered toxic, and their concentration in the plants of Ambagarh Chowki was higher than those reported in other regions such as China, Bangladesh, Brazil, Iraq, Thailand, and the USA [21,41,42,43,44,45,46].

The Kruskal–Wallis test, a non-parametric method, was employed to determine significant differences in total element content among plant species. The analysis revealed significant differences between plant species (*p* = 0.023). Further examination showed that rice grain and wheat grain differed significantly from rice root (*p* = 0.043) and wheat root (*p* = 0.046), respectively, in terms of total element content. Additionally, maize grain exhibited statistically significant differences in total element content compared to wheat straw (*p* = 0.040), rice husk (*p* = 0.017), rice root (*p* = 0.0041), and wheat root (*p* = 0.004). These results indicate that metal deposition varied depending on the plant species and organ.

### 3.3. Comparison of Element Distribution in Various Rice Cultivars

Diverse rice varieties are cultivated to enhance productivity, biological value, and nutraceutical properties, and improve adaptability to changing climate conditions, such as reduced precipitation [47]. These cultivars (*Luchai*, *DRR51*, *RI64*, *MTU1010*, *Sarna*, and *Sonam*) have maturation periods of 100 to 145 days and grain yields ranging from 32 to 50 Q/ha (quintals per hectare) [48,49].

**Figure 3:**
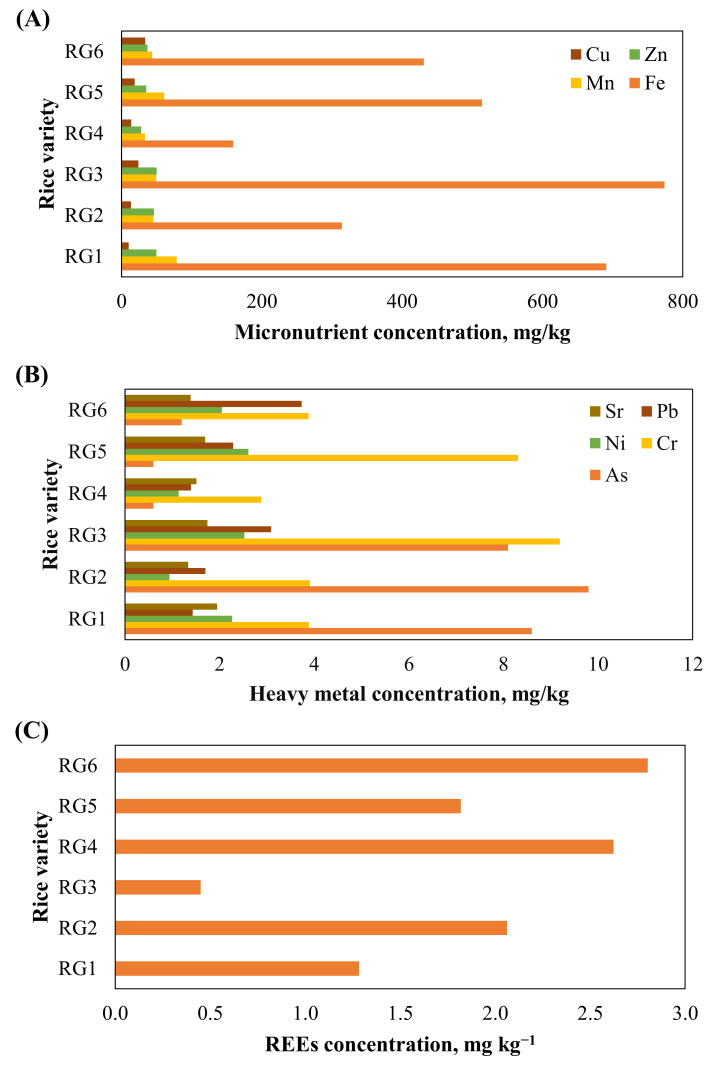
Graphical representation of multielement content in various rice varieties: (**A**) Cu, Zn, Mn, and Fe contents; (**B**) Sr, Pb, Ni, Cr, and As contents; (**C**) REEs content in grain. RG1, RG2, RG3, RG4, RG5, and RG6 stand for *Luchai*, *DRR51*, *RI64*, *MTU1010*, *Sarna*, and *Sonam* rice grain, respectively.

The distribution of the 52 elements in the grains of the six rice cultivars depended upon plant morphology, environmental factors, and uptake mobility [50], and their concentrations are shown in Table S2 and Figure 3A–C. The sum of the total concentration of these elements in the rice cultivars ranged from 11,670 to 16,641 mg/kg, with the maximum value observed in the *Sarna* cultivar grain (RG5), followed by *Luchai* (RG1). The maximum concentrations of Fe and Zn, Mn, and Cu were found in *RI64* (RG3), *Luchai*, and *Sonam* (RG6) cultivar grains (Figure 3A), respectively, whereas the maximum concentrations of As, Pb, Cr, Ni, and Sr were observed in *DRR51* (RG2), *Sonam*, *RI64*, *Sarna*, and *Luchai* cultivar grains, respectively (Figure 3B). A remarkable concentration of REEs was recorded in *Sonam* cultivar grains, followed by *MTU1010* (RG4), as shown in Figure 3C. The mean concentration (*n* = 18) of elements was observed in the following decreasing order: P (4827 ± 1094) > K (3949 ± 655) > Mg (2292 ± 546) > Ca (938 ± 428) > Al (528 ± 299) > Fe (480 ± 327) > Mn (52.0 ± 20.5) > Zn (41.0 ± 11.3) > Na (40.0 ± 38.7) > Ti (37.7 ± 24.7) > Cu (19.0 ± 9.4) > Rb (8.8 ± 3.4) > Ba (8.2 ± 4.7) > Cr (5.3 ± 4.2) > As (4.8 ± 4.1) > Pb (2.3 ± 1.0) > Ni (1.9 ± 1.3) > Sr (1.6 ± 0.3) > V (1.0 ± 0.7) > Sc (0.96 ± 0.122) > Ce (0.94 ± 0.67) Mo (0.60 ± 0.17) > Co (0.53 ± 0.57) > Sn (0.34 ± 0.14) > La (0.29 ± 0.22) > Nd (0.29 ± 0.18) > Li (0.22 ± 0.12) > Y (0.21 ± 0.12) > Ga (0.16 ± 0.01) > Th (0.13 ± 0.07) > Nb (0.11 ± 0.07) > Pr (0.08 ± 0.05) > Sm (0.06 ± 0.04) > Se (0.05 ± 0.01) > Ge (0.04 ± 0.02) > Dy (0.043 ± 0.026) > Gd (0.041 ± 0.025) > W (0.031 ± 0.010) > U (0.026 ± 0.013) > Er (0.025 ± 0.015) > Yb (0.023 ± 0.013) > Sb (0.020 ± 0.009) > Cd (0.019 ± 0.018) > Be (0.016 ± 0.011) > Eu (0.013 ± 0.008) > Ho (0.009 ± 0.005) > Te (0.009 ± 0.006) > Tb (0.007 ± 0.005) > Tl (0.005 ± 0.003) > Bi (0.004 ± 0.002) > Tm (0.004 ± 0.002) > Lu (0.003 ± 0.002).

The highest content of 25 elements (Ba, Bi, Cd, Co, Ge, Mg, Mn, Mo, Nb, Ni, Se, Sr, Ti, Tl, Y, Zn, La, Ce, Pr, Nd, Sm, Eu, Gd, Tb, and Tm) was found to accumulate in the late-maturing *Luchai* cultivar, possibly due to its higher yield. Similarly, 16 elements (Al, Be, Cr, Fe, Ga, Li, Sc, Th, U, V, W, Dy, Ho, Er, Tm, and Yb) accumulated the most in the high-yielding and high-quality *RI64* cultivar, 5 elements (Ca, K, Na, P, and Te) were found in the *Sarna* cultivar, 5 elements (Cu, Pb, Rb, Sb, and Sn) were found in the *Sonam* cultivar, and As was found in the *DRR37* cultivar.

### 3.4. Comparison of Distribution of Elements in Rice, Wheat, and Maize Grains

**Figure 4:**
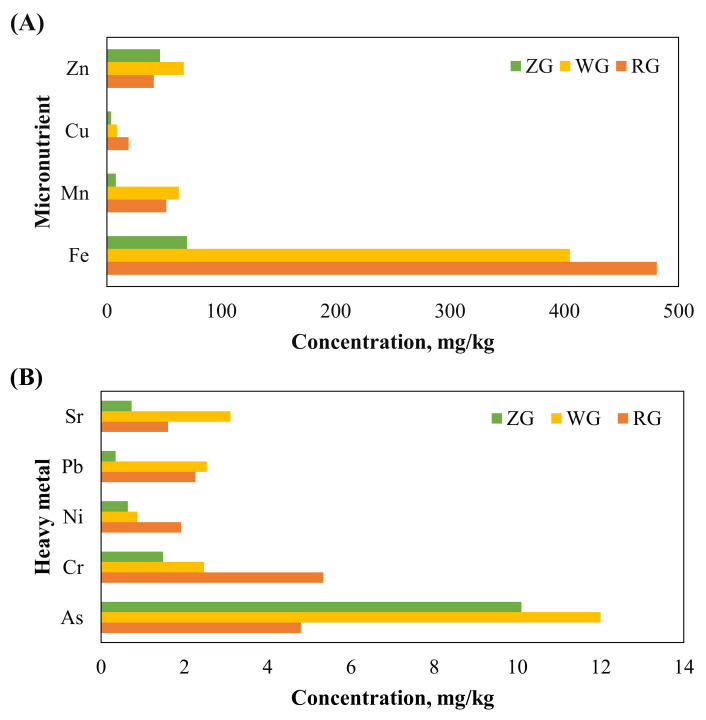
Graphical representation of multielement content variation in rice (RG), wheat (WG), and maize (MG) grains: (**A**) Zn, Cu, Mn, and Fe contents; (**B**) Sr, Pb, Ni, Cr, and As content.

The total content of the 52 elements in rice, wheat, and maize grains (Figure 4A,B) ranged from 10,249 to 13,238 mg/kg, with the maximum value observed in rice. Forty elements (Al, Ca, Co, Cr, Cu, Fe, Ga, Li, Mg, Mo, Na, Nb, Ni, P, Pb, Rb, Sb, Sc, Sn, Te, Th, Tl, U, V, W, Y, La, Ce, Pr, Nd, Sm, Eu, Gd, Tb, Dy, Ho, Er, Tm, Yb, and Lu) exhibited their highest values in the rice cultivars. In contrast, the remaining 12 elements showed higher concentrations in the wheat cultivars.

### 3.5. Distribution of Elements in Straw, Husk, and Root

The husk, straw, and root of the grains are used for agricultural activities, animal feed, energy generation, construction materials, carbon sequestration, phytoremediation of heavy metals, etc. [3,51]. Higher concentrations (approximately 1.5, 2.8, and 4.9-fold mass excess) of all elements were observed in the straw, husk, and root, respectively, compared to the grain, following a decreasing sequence: root > straw> husk > grain. A similar pattern of translocation of nutrients and heavy metals in rice plants has been previously reported [52,53].

**Figure 5:**
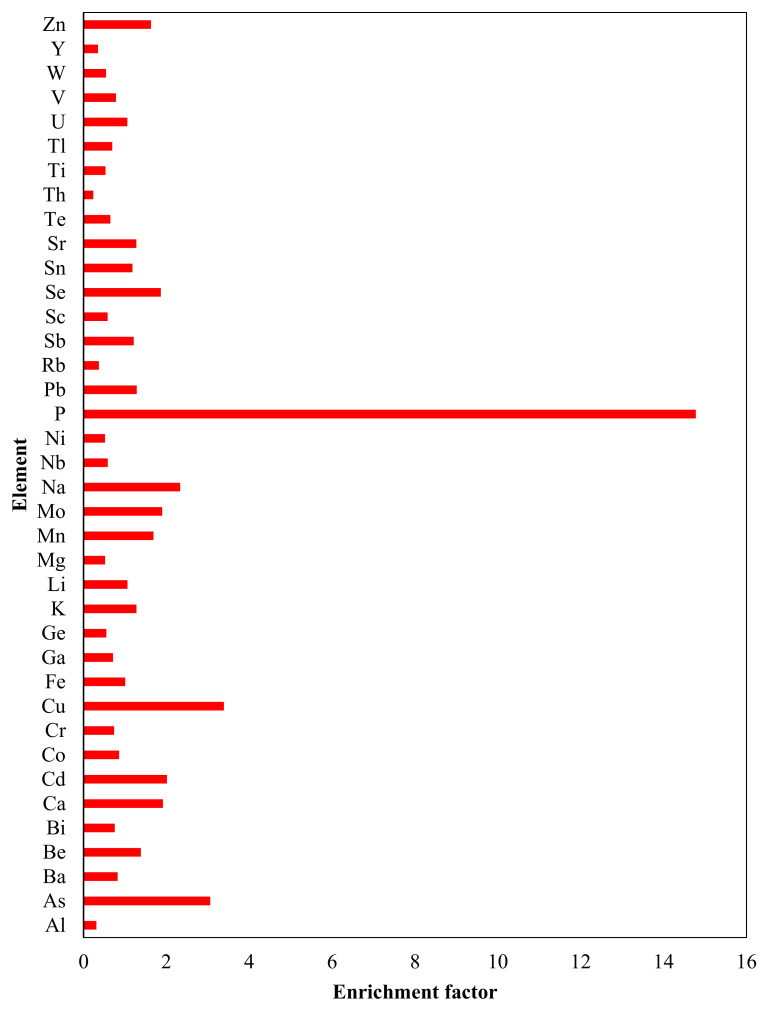
Graphical representation of E_f_ value for the studied elements by the rice root.

The maximum concentration of most elements was observed in the root. However, the maxima of P, K, Mg, Mn, and Pb were detected in either the straw or husk. The ΣREEs content in the rice husk, straw, and root ranged from 2.53 to 39.49 mg/kg. In rice plants, the root had a transfer value (Tf) of 0.20 for ΣREEs. The transfer values of other elements by the rice root is presented in Figure 5.

### 3.6. Transfer Factor

If the enrichment factor (E_f_) is utilized, which compares the element’s enrichment in the sample relative to a reference element (considering both sample and background concentrations), the resulting values would be 1.88 times higher than the T_f_ value. This discrepancy arises from the lower Al content in the study area’s soil compared to the standard value.

The plant uptake of elements from soil solution depends on various chemical environmental and bioavailability factors [54]. The transfer factor (T_f_) and enrichment factor (E_f_) values for rice and wheat grain, husk, straw, and root are presented in Table S3 and Figure 5. The T_f_ value was found to increase from grain to root.

Phosphorus was transferred to all parts of these plants, with T_f_ and E_f_ values ranging from 7.97 to 61.67 (14.98 to 115.94). Conversely, elements such as As, Na, K, Ca, Cu, Cd, Mn, Mo, and Se were poorly transferred to other parts of the plants (i.e., husk, straw, and grain). Potassium and Cu were highly enriched in the WS. Arsenic was transferred and enriched highly (1.88 and 3.53) in the rice root. Calcium, Cd, Pb, and Sn, were highly enriched in the wheat straw and root. Elements such as Be, Fe, Mo, Sb, Se, Sr, and U were highly enriched in the rice root (Figure 5). Manganese was highly transferred to the rice straw.

### 3.7. Translocation Factor

The uptake of elements from the root throughout the plant is carried out via the xylem and phloem [55]. The translocation factors (T_r_) of rice and wheat are summarized in Table S4. The elements from the soil are stored in the roots, from where they are transported to other parts of the plant. Their T_r_ values were found in the following decreasing order: straw > husk > grain. The T_r_ values of three elements, Cr, Cu, and Li, varied from 1.08 to 3.53 in the RH due to transport and environmental inputs (Table S4). In turn, a higher translocation of metals such as Cu, Li, Sb, Sn, and Zn was recorded in the WS. In the wheat grain, the T_r_ ratio for Sb and Zn was 1.15. In the wheat straw, the T_r_ value for five elements, Cu, Li, Sb, Sn, and Zn, varied from 1.20 to 9.6, with additional arial accumulation and contamination.

### 3.8. Toxicity

The health assessment index values, such as average total dose (ATD), chronic daily intake (CDI), hazard quotient (HQ), and cancer risk (CR), for toxic elements (As, Cr, Ni, Pb, and Cd) are listed in Table S5. Their highest values were recorded for wheat. The total hazard index (ΣHI) and total cancer risk (ΣTCR) values for rice, maize, and wheat were calculated to be 155, 285, and 346 and 0.117, 0.142, and 0.187, respectively.

## 4. Discussion

A good agreement between the observed and certified data was obtained for all elements; the recoveries obtained ranged from 91% to 122% for the soil (NCS DC 77302) and from 81% to 108% for the citrus leaves (NCS ZC73018) reference material, respectively.

A higher concentration of most of the 52 elements in the rice grain was observed, probably due to the waterlogged conditions in which the plant is cultivated (Table 1). However, a higher concentration of As (10.1–12.0 mg/kg) in the maize and wheat grain was recorded. The rice (Ca, Mg, Fe, Mo, Na) and wheat (K, Mn, Se, and Z) grains were rich in nutrient contents. Elements such as Na and Te; and Na, Tl, Te, La, Tb, Ho, Tm, and Lu were not detectable in the wheat and maize grains, respectively (Table 1).

The correlation patterns in the rice and wheat plant parts showed similarities, suggesting similar uptake patterns and potential links to the soil’s elemental composition. The degree of correlation of elements was found to increase from grain to root: RG < RH < RS < Rr. Thirty-two elements (Al, Ba, Be, Bi, Cu, Ga, Li, Sn, Nb, Pb, Sb, Th, Ti, Tl, U, V, W, Y and REEs), 39 elements (Al, Be, Bi, Ca, Cr, Ga, Ge, K, Li, Nb, Ni, Rb, Sc, Se, Sn, Sr, Te, Th, Ti, Tl, U, V, W, Y, Zn and REEs), and 46 elements (Al, As, Ba, Be, Bi, Ca, Cd, Co, Cr, Fe, Ga, Ge, Li, Mn, Mo, Nb, Ni, P, Pb, Rb, Sc, Se, Sn, Sr, Te, Th, Ti, Tl, U, V, W, Y and REEs) were well correlated in the rice grain/husk, straw and root, respectively.

Thirty-two (Al, Ba, Be, Bi, Co, Fe, Ga, Ge, Li, Nb, Ni (except with W, Zn and Ce) Sc, Th, Ti, Tl, U, V, W, Y, La, Ce, Pr, Nd, Sm, Eu, Gd, Tb, Dy, Ho, Er, Tm, and Yb), and thirty-nine elements (Al, Ba, Be, Bi, Co, Cr, Cu, Fe, Ge, Ga, Li, K, Mo, Nb, Ni, Sc, Se, Sr, Th, Ti, Tl, U, V, W, Y, La, Ce, Pr, Nd, Sm, Eu, Gd, Tb, Dy, Ho, Er, Tm, Yb and Lu) showed strong correlations (ranging from 0.68 to 1.0) within the studied rice and wheat grains, respectively. However, no correlation of these elements in the maize grain was found. In addition, a good correlation (0.70–0.98) among specific elements such as As, Ba, Bi, Zn, Ce, Cr, Ni, Sc, Th, U, Cu, Pb, Sb, Sn, K, Mg, P, Te, Mo, Se, Sr, Na, and Ca in rice grain was recorded. No correlation of Cd and Rb with other elements in the rice grain was observed.

Factor analysis was applied to the grain’s dataset consisting of 39 cases × 29 metals and provided insights into the primary sources of elemental contamination. Three factors were obtained (Table S6). The first factor, explaining 61.6% of the total variance, was dominated by REEs, Fe, Li, Al, Be, Bi, Co, Ni, and As. This suggests that the parent soil material is a significant contributor to the elemental profile of the grains [56,57]. The geological characteristics of the Ambagarh Chowki region, known for its mineral-rich deposits, likely play a crucial role in this contamination.

The second factor, accounting for 13.98% of the variance and loaded with Cu, Pb, and K, points to anthropogenic influences. The presence of Pb, a known tracer for vehicle exhaust, indicates contributions from vehicular emissions [58]. Additionally, the high loading of Cu suggests inputs from agricultural activities as copper-based pesticides and fertilizers are commonly used in crop production [59,60,61].

The third factor, explaining 7.3% of the variance and dominated by Mg, Mo, and P, likely represents natural soil nutrient sources. However, the elevated levels of these elements may also reflect intensive agricultural practices and the application of phosphate fertilizers [62].

These findings underscore the complex nature of contamination in the study area, where both natural geogenic sources and human activities contribute to the elevated levels of potentially harmful elements in agricultural soils and, consequently, in food crops.

Given that As was the main contributor to the HI and CR values, the health risks associated with cereals primarily arise from natural geogenic sources (the parent soil material). It is worth noting that the concentration in the soil analyses reported herein was higher than those reported in other locations [22,23,24,37,38]. The same applies to the mineral concentrations in the grains of the studied area, which were higher than those reported in other regions for several elements, including As, Cr, Cd, Zn, Pb, Ba, and U [21,41,42,43,44,45,46,63,64].

A toxicity assessment was imperative, particularly considering that the three grains—rice, wheat, and maize—are fundamental staples in the country’s diet, while their straw and husk serve as vital feed for domestic livestock such as cows, buffaloes, and goats. With regard to hazardous elements (Pb, Ni, V, Cu, Zn, Fe, Mn, Sb, Ba, Be, Li, Sr, Ti, U, Co, Se, and Sn) concentrations, a significant majority surpassed their respective permissible limits (0.30, 0.1, 0.03, 10, 30, 500, 0.02, 0.002, 0.002, 0.004, 0.01, 0.001, 0.0004, 0.03, 0.01 and 0.01 mg/kg) in the cereals cultivated in Ambagarh Chowki [65,66,67,68,69].

The health risk assessment revealed concerning levels of toxic elements in all studied cereals. Elements such as As, Ba, Co, Li, Ni, Pb, Se, Sn, Sr, U, and V accumulated beyond their respective permissible limits of 0.2, 0.002, 0.01, 0.004, 0.1, 0.03, 0.01, 0.01, 0.0004, and 0.03 mg/kg in grains, straws, and husks [65,66,67,68,69]. Other elements, such as Cr, Cu, Tl, and REEs, exceeded their prescribed limits of 2.3, 10, 0.001, and 0.7 mg/kg, respectively, in rice grain, straw, and husk [70]. Elements such as Fe and Zn accumulated beyond the limits of 500 and 50 mg kg^−1^, respectively, in the straw and husk. The aluminum content accumulated in rice, higher than in wheat and maize (Table 1), also exceeded the provisional tolerable weekly intake (PTWI) (7.0 mg/kg/body weight) [40]. This widespread contamination poses significant health risks to both humans and livestock, as these plant parts are used for food and animal feed.

The HI values of <1, >1, and ≥10 were reported as no adverse, non-carcinogenic, and chronic toxic effects, respectively [71].

As for the estimated toxicity indices (Table S5), the HI and CR values for As, Cd, Cr, Ni, and Pb in the study area were several folds higher than those reported in China, India, Iran, and Malaysia [72,73].

The study findings suggest that rice may be a safer food option compared to wheat and maize. The hazard quotient for rice, wheat, and maize was far above 1, which suggests a potential non-cancer health risk [74]. Moreover, the cancer risk for these grains exceeded the acceptable limit: 1 × 10^−4^ [75]. These findings are particularly alarming given that these grains form the staple diet for the local population.

### 4.1. Implications for Food Safety and Agriculture

This study found that wheat and maize posed greater health risks compared to rice, despite rice showing higher total elemental accumulation. This counterintuitive result underscores the importance of considering not just total elemental content, but also the specific toxic elements and their relative concentrations when assessing health risks.

The identification of rice varieties with a lower accumulation of toxic elements (*MTU1010*, *Sarna*, and *Sonam*) provides a potential avenue for mitigating health risks in the short term. Promoting the cultivation of these varieties could help reduce exposure to toxic elements while longer-term remediation strategies are developed and implemented.

However, it is important to note that even these “safer” varieties still exceeded permissible limits for several toxic elements, highlighting the severity of soil contamination in the region. This emphasizes the urgent need for comprehensive soil remediation efforts and the development of agricultural practices that minimize elemental uptake by food crops.

### 4.2. Limitations and Future Directions

While this study provides valuable insights into the elemental contamination of cereals in Ambagarh Chowki, several limitations should be acknowledged. Our sampling was limited to a single growing season, and temporal variations in elemental accumulation were not assessed. Additionally, the study did not investigate the speciation of elements, which can significantly influence their bioavailability and toxicity.

Future research should focus on long-term monitoring of elemental accumulation in various crop varieties under different agricultural management practices. Studies on the effectiveness of soil amendments and phytoremediation techniques in reducing elemental uptake by crops would be particularly valuable. Furthermore, investigating the potential for dietary diversification and food processing methods to reduce exposure to toxic elements could provide practical solutions for improving food safety in the region.

This study highlights the critical need for integrated approaches to address the complex issue of elemental contamination in agricultural systems. Combining agronomic strategies, soil remediation techniques, and public health interventions will be essential for ensuring food safety and protecting human health in areas affected by severe soil pollution, such as Ambagarh Chowki.

## 5. Conclusions

Grains of rice, wheat, and maize, which are fundamental staples in India’s diet, accumulated 52 elements from the heavily contaminated soils of Ambagarh Chowki. Rice grains exhibited the highest total mineral content followed by wheat and maize. Within the rice plant, translocation occurred in increasing order: straw > husk > grain. Concerning levels of toxic elements, As, Ba, Co, Li, Ni, Pb, Se, Sn, Sr, U, and V, exceeded the recommended limits. Health risk assessment revealed a hazard index (ΣHQ_i_) far above 1 for rice, wheat, and maize grains due to As, Cr, Ni, Pb, and Cd contents, indicating potential non-cancer risks. Moreover, the cancer risk (ΣCR) posed by these grains surpassed the acceptable limit of 1 × 10^−4^. Rice emerged as a relatively safer option compared to wheat and maize, with certain varieties (*MTU 1010*, *Sarna*, and *Sonam*) showing a lower accumulation of toxic elements. These findings underscore the urgent need for soil remediation efforts and careful selection of crop varieties in this region. Future research should focus on developing effective management practices to reduce heavy metal uptake by crops and mitigate associated health risks. Additionally, long-term monitoring of element accumulation in various crop varieties could inform agricultural strategies to improve food safety in contaminated areas.

## Figures and Tables

**Table 1:** Distribution of elements (mg kg^−1^) in rice (*n* = 18), wheat (*n* = 3), and maize (*n* = 3) grain, rice straw (*n* = 3), rice husk (*n* = 3), wheat husk (*n* = 3), rice root (*n* = 3), and wheat root (*n* = 3).

Sample	Al	As	Ba	Be	Bi	Ca	Cd
RG	528 ± 299	4.8 ± 4.1	8.2 ± 4.7	0.015 ± 0.011	0.004 ± 0.002	938 ± 428	0.019 ± 0.018
WG	431 ± 230	12.0 ± 0.8	12.9 ± 3.2	0.009 ± 0.007	0.005 ± 0.001	907 ± 46	0.05 ± 0.01
ZG	49.1 ± 7.4	10.1 ± 0.3	3.0 ± 0.5	ND	0.002 ± 0	402 ± 65	0.005 ± 0.002
RH	770 ± 183	11.8 ± 0.8	13.4 ± 1.7	0.02 ± 0.01	0.016 ± 0.01	1448 ± 98	0.021 ± 9.005
WS	1628 ± 878	15.3 ± 2.8	117.8 ± 4.8	0.07 ± 0.030	0.029 ± 0.012	4933 ± 158	0.189 ± 0.099
RS	2835 ± 917	21.3 ± 5.1	87.2 ± 13.9	0.13 ± 0.06	0.027 ± 0.009	6098 ± 305	0.132 ± 0.072
Rr	6832 ± 3311	79.9 ± 34.9	222 ± 114	0.73 ± 0.38	0.119 ± 0.051	10,293 ± 4175	0.179 ± 0.075
Wr	3785 ± 1078	27.1 ± 2.2	180 ± 53	0.38 ± 0.11	0.061 ± 0.020	7087 ± 643	0.340 ± 0.212
**Sample**	**Co**	**Cr**	**Cu**	**Fe**	**Ga**	**Ge**	**K**
RG	0.53 ± 0.57	5.34 ± 4.18	19.0 ± 9.4	481 ± 327	0.16 ± 0.10	0.038 ± 0.019	3949 ± 655
WG	0.32 ± 0.14	2.47 ± 1.03	8.7 ± 1.4	405 ± 217	0.13 ± 0.08	0.027 ± 0.014	4769 ± 970
ZG	0.08 ± 0.20	1.49 ± 0.32	3.6 ± 0.4	70 ± 12	0.017 ± 0.004	0.005 ± 0.002	4758 ± 499
RH	0.62 ± 0.13	68.1 ± 11.4	200 ± 186	1393 ± 348	0.25 ± 0.05	0.179 ± 0.042	6318 ± 1250
WS	1.31 ± 0.84	19.7 ± 7.1	357 ± 213	2110 ± 1547	0.63 ± 0.45	0.190 ± 0.052	21,403 ± 3718
RS	3.8 ± 0.7	21.5 ± 5.6	59.9 ± 3.5	3671 ± 1449	1.30 ± 0.55	0.305 ± 0.107	12,293 ± 1916
Rr	14.0 ± 7.6	69.0 ± 24.8	83.1 ± 31.6	22,923 ± 12,810	6.44 ± 3.59	0.898 ± 0.421	8233 ± 3037
Wr	12.1 ± 4.5	31.9 ± 5.9	37.1 ± 0.5	14,442 ± 4189	3.90 ± 1.17	0.549 ± 0.128	8441 ± 2493
**Sample**	**Li**	**Mg**	**Mn**	**Mo**	**Na**	**Nb**	**Ni**
RG	0.22 ± 0.10	2292 ± 546	52 ± 21	0.60 ± 0.17	34.8 ± 38.7	0.11 ± 0.07	1.92 ± 1.35
WG	0.16 ± 0.10	1402 ± 348	63 ± 21	0.36 ± 0.07	ND	0.10 ± 0.05	0.87 ± 0.28
ZG	0.03 ± 0.02	1378 ± 126	7.9 ± 0.9	0.29 ± 0.04	ND	0.013 ± 0.001	0.64 ± 0.03
RH	0.28 ± 0.07	2389 ± 414	217 ± 16	0.61 ± 0.02	ND	0.18 ± 0.05	3.49 ± 0.31
WS	0.77 ± 0.54	1806 ± 351	122 ± 49	0.42 ± 0.08	290 ± 132	0.47 ± 0.31	2.85 ± 1.73
RS	1.67 ± 0.57	2581 ± 361	1230 ± 557	0.444 ± 0.104	2956 ± 465	1.03 ± 0.53	4.37 ± 1.22
Rr	7.70 ± 5.43	1937 ± 641	1007 ± 883	0.76 ± 0.09	3974 ± 1442	5.07 ± 2.79	21.20 ± 122.
Wr	5.15 ± 1.65	1384 ± 175	540 ± 248	0.41 ± 0.07	995 ± 315	2.62 ± 0.66	17.40 ± 5.39
**Sample**	**P**	**Pb**	**Rb**	**Sb**	**Sc**	**Se**	**Sn**
RG	4827 ± 1094	2.3 ± 1.0	8.8 ± 3.4	0.02 ± 0.01	1.0 ± 0.2	0.06 ± 0.01	0.34 ± 0.14
WG	3989 ± 776	2.5 ± 2.2	2.7 ± 1.5	0.011 ± 0.003	0.77 ± 0.15	0.07 ± 0.03	0.28 ± 0.13
ZG	3504 ± 321	0.35 ± 0.08	7.8 ± 1.5	0.002 ± 0.002	0.48 ± 0.01	0.04 ± 0.01	0.15 ± 0.02
RH	4288 ± 717	25.2 ± 22.6	11.3 ± 3.43	0.16 ± 0.13	10.0 ± 2.9	0.049 ± 0.004	2.65 ± 2.25
WS	801 ± 136	46.5 ± 28.3	6.0 ± 2.4	0.29 ± 0.16	6.9 ± 0.59	0.08 ± 0.01	4.80 ± 2.82
RS	956 ± 202	8.3 ± 0.8	8.4 ± 2.8	0.09 ± 0.02	12.7 ± 1.0	0.08 ± 0.02	0.95 ± 0.10
Rr	929 ± 470	20.2 ± 7.6	16.4 ± 7.7	0.51 ± 0.16	13.4 ± 6.5	0.21 ± 0.03	2.20 ± 0.65
Wr	982 ± 20	8.9 ± 2.0	10.2 ± 1.7	0.20 ± 0.05	11.0 ± 2.49	0.16 ± 0.07	1.37 ± 0.44
**Sample**	**Sr**	**Te**	**Th**	**Ti**	**Tl**	**U**	**V**
RG	1.61 ± 0.32	0.006 ± 0.006	0.13 ± 0.07	37.5 ± 24.7	0.005 ± 0.003	0.026 ± 0.013	1.01 ± 0.73
WG	3.11 ± 0.54	ND	0.09 ± 0.05	40.6 ± 22.5	0.003 ± 0.002	0.016 ± 0.008	0.91 ± 0.56
ZG	0.73 ± 0.21	ND	0.008 ± 0.002	4.8 ± 0.9	ND	0.003 ± 0.001	0.07 ± 0.01
RH	6.05 ± 1.15	0.005 ± 0.008	0.196 ± 0.059	57.4 ± 17.9	0.009 ± 0.002	0.044 ± 0.010	1.32 ± 0.39
WS	26.3 ± 1.6	0.005 ± 0.008	0.35 ± 0.13	204 ± 143	0.023 ± 0.011	0.120 ± 0.083	4.58 ± 0.33
RS	32.1 ± 2.0	0.008 ± 0.007	0.77 ± 0.32	309 ± 130	0.047 ± 0.021	0.497 ± 0.037	9.12 ± 2.85
Rr	53.5 ± 19.0	0.022 ± 0.012	2.04 ± 0.87	1497 ± 1063	0.223 ± 0.115	1.77 ± 1.09	46.2 ± 29.4
Wr	37.6 ± 10.4	ND	0.62 ± 0.26	1151 ± 389	0.115 ± 0.035	0.65 ± 0.19	36.8 ± 9.3
**Sample**	**W**	**Y**	**Zn**	**La**	**Ce**	**Pr**	**Nd**
RG	0.03 ± 0.01	0.21 ± 0.12	41.1 ± 11.3	0.29 ± 0.22	0.94 ± 0.67	0.08 ± 0.05	0.29 ± 0.18
WG	0.032 ± 0.007	0.16 ± 0.09	67.3 ± 2.2	0.20 ± 0.14	0.69 ± 0.37	0.06 ± 0.03	0.24 ± 0.13
ZG	0.016 ± 0.003	0.017 ± 0.016	46.6 ± 4.9	ND	0.06 ± 0.1	0.006 ± 0	0.024 ± 0
RH	0.107 ± 0.016	0.32 ± 0.094	65.1 ± 11.9	0.46 ± 0.14	1.18 ± 0.034	0.12 ± 0.03	0.45 ± 0.11
WS	0.11 ± 0.06	0.70 ± 0.37	71.6 ± 18.1	1.10 ± 0.64	2.70 ± 1.46	0.29 ± 0.18	1.09 ± 0.68
RS	0.18 ± 0.05	1.63 ± 0.64	48.5 ± 9.9	2.49 ± 1.32	5.23 ± 2.70	0.62 ± 0.33	2.27 ± 1.89
Rr	0.70 ± 0.41	4.57 ± 1.28	76.6 ± 2.5	7.48 ± 1.57	16.5 ± 4.6	1.97 ± 0.58	7.56 ± 2.36
Wr	0.45 ± 0.10	1.79 ± 0.65	59.0 ± 5.9	3.14 ± 0.86	9.92 ± 1.91	0.99 ± 0.21	3.88 ± 0.83
**Sample**	**Sm**	**Eu**	**Gd**	**Tb**	**Dy**	**Ho**	**Er**
RG	0.060 ± 0.037	0.013 ± 0.008	0.04 ± 0.03	0.007 ± 0.005	0.04 ± 0.03	0.009 ± 0.005	0.025 ± 0.015
WG	0.047 ± 0.028	0.012 ± 0.006	0.031 ± 0.019	0.006 ± 0.003	0.032 ± 0.018	0.007 ± 0.004	0.019 ± 0.010
ZG	0.006 ± 0	0.002 ± 0	0.003 ± 0.001	ND	0.003 ± 0.001	ND	0.002 ± 0
RH	0.091 ± 0.021	0.019 ± 0.004	0.059 ± 0.016	0.011 ± 0.003	0.062 ± 0.019	0.013 ± 0.004	0.036 ± 0.010
WS	0.231 ± 0.139	0.067 ± 0.028	0.15 ± 0.09	0.03 ± 0.02	0.16 ± 0.10	0.03 ± 0.02	0.09 ± 0.06
RS	0.522 ± 0.226	0.108 ± 0.035	0.336 ± 0.142	0.065 ± 0.030	0.366 ± 0.142	0.075 ± 0.036	0.216 ± 0.103
Rr	1.591 ± 0.55	0.323 ± 0.142	1.01 ± 0.33	0.20 ± 0.06	1.18 ± 0.38	0.24 ± 0.08	0.70 ± 0.23
Wr	0.795 ± 0.178	0.198 ± 0.046	0.49 ± 0.11	0.10 ± 0.02	0.57 ± 0.14	0.12 ± 0.08	0.33 ± 0.08
**Sample**	**Tm**	**Yb**	**Lu**	**ΣElement**	**ΣREEs**	**ΣHREEs**	**ΣLREEs**
RG	0.004 ± 0.002	0.023 ± 0.013	0.003 ± 0.002	13,238 ± 2569	1.84 ± 1.26	1.69 ± 1.15	0.16 ± 0.09
WG	0.003 ± 0.001	0.016 ± 0.010	0.003 ± 0.001	12,125 ± 2437	1.37 ± 0.77	1.25 ± 0.70	0.12 ± 0.06
ZG	ND	0.002 ± 0	ND	10,249 ± 973	0.11 ± 0.01	0.10 ± 0.01	0.01 ± 0
RH	0.005 ± 0.002	0.033 ± 0.010	0.005 ± 0.002	17,304 ± 2395	2.53 ± 0.72	2.31 ± 0.65	0.22 ± 0.07
WS	0.013 ± 0.008	0.086 ± 0.058	0.013 ± 0.009	33,987 ± 2142	6.05 ± 3.45	5.48 ± 3.12	0.58 ± 0.37
RS	0.031 ± 0.015	0.192 ± 0.094	0.027 ± 0.013	33,283 ± 4367	14.22 ± 1.43	12.91 ± 5.79	1.31 ± 0.60
Rr	0.10 ± 0.03	0.61 ± 0.21	0.08 ± 0.03	58,413 ± 23,420	39.49 ± 11.14	35.38 ± 9.80	4.12 ± 1.33
Wr	0.05 ± 0.01	0.29 ± 0.07	0.04 ± 0.01	39,313 ± 871	20.90 ± 4.51	18.93 ± 3.83	1.97 ± 0.47

RG, WG, ZG, RS, WS, RH, Rr, Wr, and ND stand for rice grain, wheat grain, maize grain, rice straw, wheat straw, rice husk, rice root, wheat root, and not detectable, respectively.

## Data Availability

The data are available in the text and Supplementary Materials. In addition, further information related to this MS is available from the corresponding author, K.S. Patel: patelkhageshwarsingh@gmail.com.

## Supplementary Materials

The following supporting information can be downloaded at: https://www.mdpi.com/article/10.3390/toxics13010056/s1. Table S1: ICP-MS experimental conditions; Table S2: Distribution of elements in the grain (n = 3) of rice varieties cultivated in Ambagarh Chowki, mg/kg; Table S3: Transfer factor (T_f_) values of elements in cereals cultivated in Ambagarh Chowki; Table S4: Translocation (T_r_) factors of elements in rice and wheat cultivated in Ambagarh Chowki; Table S5: Health hazard parameters of toxic elements for cereal samples; Table S6: Factor loadings indicating the contribution of elements measured in grains to each factor in the factor analysis-based source apportionment.
