# Supplementary material for: Multi-Element Exposure and Health Risks of Grains from Ambagarh Chowki, Chhattisgarh, India
Source: Toxics. 2025 Jan 14;13(1):56. doi: 10.3390/toxics13010056 (PMC11769171; doi:10.3390/toxics13010056)
Supplement: Supplementary file 1 [file toxics-13-00056-s001.zip › toxics-3320204-supplementary.pdf]

Multi-element exposure and health risks of grains from Ambagarh Chowki, Chhattisgarh, India

Bhagyashri Wakhle , Saroj Sharma, Khageshwar Singh Patel, Piyush Kant Pandey, Antonela Blažević, Željka Fiket, Sema Yurdakul, Simge Varol, Pablo Martín-Ramos, Hanan Mohamed Al-Yousef, and Ramzi Ahmed Mothana

Table.S1 Instrumental conditions of used mass spectrometer and data acquisition parameters for determination of selected elements.

Table.S2 Distribution of elements in the grain (n =3) of rice varieties cultivated in Ambagarh Chowki, mg kg.

Table.S3 Transfer factor (T_f) values of elements in cereals cultivated in Ambagarh Chowki.

Table.S4 Translocation (T_r) factors of elements in rice and wheat cultivated in Ambagarh Chowki.

Table.S5 Health hazard parameters of toxic elements for cereal samples.

Table.S6 Factor loadings indicating the contribution of elements measured in grains to each factor in the factor analysis-based source apportionment.

Table S1. Instrumental conditions of used mass spectrometer and data acquisition parameters for determination of selected elements.

Parameter	Value
ICP-QQQ	8900 Agilent, USA
RF power	1550 W
Plasma gas flow rate	15.0 L/ min
Auxiliary gas flow rate	0.90 L/ min
Sample gas flow rate	1.01 L/ min

Torch	Fassel type, 1.5 mm i.d.
Nebulizer	MicroMist (Nebulizer Sample Particle Size Tolerance - 40 µm)
Sample cone	Ni
Skimmer cone	Ni
Acquisition mode	Spectrum
No. Scans	50
Calibration	External

Table S2. Distribution of elements in the grain (n =3) of rice varieties cultivated in Ambagarh Chowki, mg/kg.

Grain	Al	As	Ba	Be	Bi	Ca	Cd	Co
RG1	706±236	8.6±0.7	13.2±3.4	0.020±0.005	0.006±0	737±39	0.017±0.008	1.25±0.61
RG2	366±228	9.8±0.5	8.7±3.1	0.014±0.013	0.004±0.002	724±107	0.013±0.002	0.26±0.13
RG3	854±99	8.1±0.4	11.4±0.72	0.022±0.004	0.005±0.001	679±99	0.014±0.003	0.67±0.30
RG4	246±119	0.57±0.02	2.0±0.7	0.006±0.003	0.002±0	1188±454	0.008±0.001	0.12±0.03
RG5	558±369	0.64±0.11	9.0±4.8	0.022±0.017	0.003±0.002	1553±326	0.035±0.028	0.68±0.73
RG6	439±130	1.2±0.96	5.0±1.7	0.013±0.006	0.004±0	746±364	0.026±0.022	0.21±0.06
**Grain**	**Cr**	**Cu**	**Fe**	**Ga**	**Ge**	**K**	**Li**	**Mg**
RG1	3.89±0.074	10.1±1.6	691±195	0.218±0.068	0.054±0.012	4397±190	0.307±0.108	2985±190
RG2	3.91±1.96	13.4±5.2	314±204	0.123±0.096	0.031±0.016	3619±423	0.146±0.094	2047±372
RG3	9.19±2.11	24.1±3.70	774±214	0.256±0.025	0.054±0.010	3471±462	0.321±0.061	1935±326
RG4	2.88±1.67	13.8±3.0	159±53	0.063±0.029	0.017±0.007	3740±331	0.139±0.064	1929±241
RG5	8.31±7.40	18.7±5.9	514±449	0.151±0.12	0.043±0.023	4798±192	0.232±0.141	2867±41

RG6	3.88±1.48	33.7±7.7	431±214	0.120±0.042	0.028±0.003	3670±786	0.144±0.027	1990±480
**Grain**	**Mn**	**Mo**	**Na**	**Nb**	**Ni**	**P**	**Pb**	**Rb**
RG1	78.8±23.7	0.757±0.179	ND	0.148±0.055	2.27±0.71	5888±287	1.43±0.24	9.18±3.50
RG2	45.4±5,2	0.575±0.003	ND	0.088±0.059	0.94±0.28	4510±739	1.70±0.56	7.56±1.53
RG3	49.6±5.1	0.637±0.203	ND	0.187±0.024	2.53±0.98	3921±791	3.09±0.46	7.01±0.25
RG4	33.4±3.0	0.630±0.061	52.0±6.4	0.037±0.011	1.14±0.48	4246±410	1.39±0.20	7.36±267
RG5	61.0±24.9	0.590±0.031	90.4±28.6	0.106±0.072	2.61±1.98	6070±77	2.29±0.75	8.77±3.73
RG6	43.6±4.4	0.430±0.187	66.8±9.2	0.088±0.034	2.05±1.62	4325±1254	3.73±0.73	13.0±2.6
**Grain**	**Sb**	**Sc**	**Se**	**Sn**	**Sr**	**Te**	**Th**	**Ti**
RG1	0.015±0.003	0.97±0.19	0.065±0.007	0.25±0.02	1.95±0.27	0.005±0.007	0.168±0.045	59.1±23.8
RG2	0.013±0.006	0.96±0.15	0.057±0.020	0.27±0.076	1.34±0.31	ND	0.102±0.063	25.5±15.8
RG3	0.027±0.008	1.12±0.11	0.051±0.009	0.39±0.05	1.75±0.11	ND	0.185±0.006	67.1±14.1
RG4	0.014±0.005	0.85±0.18	0.053±0.012	0.36±0.27	1.51±0.20	0.003±0.003	0.068±0.050	13.4±4.7
RG5	0.020±0.009	1.00±0.19	0.047±0	0.32±0.05	1.70±0.28	0.022±0.010	0.141±0.08	35.4±19.5
RG6	0.030±0.006	0.87±0.031	0.046±0.046	0.46±0.05	1.39±0.20’	0.004±0.005	0.104±0.041	25.4±4.8
**Grain**	**Tl**	**U**	**V**	**W**	**Y**	**Zn**	**La**	**Ce**
RG1	0.007±0.001	0.033±0.008	1.64±0.56	0.038±0.009	0.311±0.097	49.73±6.2	0.46±0.13	1.42±0.45
RG2	0.003±0.002	0.022±0.015	0.64±00.50	0.027±0.010	0.171±0.104	46.31±12.3	0.24±0.21	1.08±0.82
RG3	0.007±0.002	0.035±0.004	1.73±0.55	0.041±0.007	0.331±0.016	50.3±5.0	0.45±0.01	1.24±0.17
RG4	0.002±0	0.014±0.009	0.32±0.14	0.023±0.003	0.071±0.028	28.0±2.9	0.05±0.04	0.23±0.08
RG5	0.005±0.002	0.033±0.016	0.93±0.80	0.027±0.007	0.229±0.130	35.4±3.8	0.35±0.29	1.06±0.85
RG6	0.004±0.002	0.021±0.009	0.77±0.32	0.028±0.009	0.171±0.052	37.0±11.0	0.21±0.09	0.63±0.34
**Grain**	**Pr**	**Nd**	**Sm**	**Eu**	**Gd**	**Tb**	**Dy**	**Ho**
RG1	0.125±0.033	0.457±0.123	0.094±0.025	0.021±0.006	0.059±0.019	0.012±0.004	0.064±0.023	0.012±0.004
RG2	0.069±0.048	0.242±0.168	0.049±0.168	0.011±0.006	0.035±0.026	0.006±0.004	0.034±0.023	0.007±0.005
RG3	0.121±0.006	0.450±0.034	0.094±0.007	0.020±0.002	0.061±0.005	0.011±0.001	0.067±0.003	0.014±0.001
RG4	0.023±0.009	0.081±0.030	0.016±0.006	0.004±0.002	0.011±0.005	0.003±0.002	0.013±0.005	0.003±0.001
RG5	0.089±0.059	0.323±0.206	0.064±0.038	0.014±0.007	0.047±0.027	0.008±0.004	0.045±0.026	0.009±0.005

RG6	0.059±0.019	0.214±0.068	0.045±0.014	0.009±0.002	0.033±0.011	0.006±0.001	0.032±0.010	0.007±0.002
**Grain**	**Er**	**Tm**	**Yb**	**Lu**	ΣREEs	ΣHREEs	ΣLREEs	ΣLREEs/ΣHREEs
RG1	0.036±0.014	0.005±0.002	0.033±0.009	0.003±0.002	2.80±0.83	2.58±0.76	0.23±0.08	11.7±1.7
RG2	0.021±0.013	0.003±0.002	0.018±0.011	0.003±0.001	1.82±1.36	1.69±1.28	0.13±0.08	12.2±2.1
RG3	0.040±0.004	0.005±0.001	0.036±0.002	0.005±0.001	2.62±0.23	2.38±0.23	0.24±0.02	9.8±0.9
RG4	0.008±0.004	0.001±0	0.008±0.003	0.005±0	0.45±0.18	0.40±0.16	0.050±0.02	8.8±1.0
RG5	0.026±0.016	0.004±0.002	0.024±0.014	0.003±0.002	2.06±1.54	1.90±1.45	0.16±0.10	11.0±2.8
RG6	0.019±0.007	0.003±0.001	0.018±0.007	0.003±0.001	1.28±0.54	1.16±0.050	0.12±0.04	9.3±1.1

RG1, RG2, RG3, RG4, RG5, and RG6 stand for Luchai, DRR51, RI64, MTU1010, Sarna, and Sonam rice grain, respectively.

Table S3. Transfer factor (T_f) values of elements in cereals cultivated in Ambagarh Chowki.

**Sample**	**Al**	**As**	**Ba**	**Be**	**Bi**	**Ca**	**Cd**	**Co**	**Cr**	**Cu**	**Fe**	**Ga**	**Ge**
RG	0.01	0.01	0.00	0.01	0.01	0.12	0.05	0	0.03	0.30	0	0	0.01
RH	0.02	0.28	0.03	0.02	0.06	0.15	0.14	0.02	0.59	4.18	0.03	0.02	0.06
RS	0.07	0.51	0.17	0.13	0.09	0.64	0.79	0.13	0.19	1.31	0.09	0.08	0.10
Rr	0.16	1.88	0.42	0.71	0.38	1.05	1.07	0.46	0.64	1.89	0.55	0.36	0.29
WG	0.01	0.26	0.03	0.01	0.02	0.13	0.28	0.01	0.03	0.16	0.01	0.01	0.01
WS	0.04	0.33	0.23	0.07	0.13	0.73	1.21	0.05	0.28	5.53	0.04	0.04	0.06
Wr	0.08	0.58	0.35	0.40	0.27	1.06	2.24	0.37	0.42	0.65	0.26	0.21	0.18
**Sample**	**K**	**Li**	**Mg**	**Mn**	**Mo**	**Na**	**Nb**	**Ni**	**P**	**Pb**	**Rb**	**Sb**	**Sc**
RG	0.31	0.01	0.31	0.03	0.84	0.02	0.00	0.02	54.00	0.05	0.09	0.02	0.02
RH	0.52	0.02	0.39	0.21	0.81	0.00	0.01	0.05	61.67	0.82	0.14	0.20	0.24
RS	1.01	0.12	0.39	1.02	0.59	0.94	0.06	0.07	10.79	0.28	0.10	0.12	0.30
Rr	0.66	0.55	0.30	0.85	1.01	1.23	0.30	0.34	9.75	0.66	0.19	0.64	0.32

WG	0.47	0.01	0.30	0.06	0.36	0.00	0.01	0.01	45.41	0.10	0.04	0.01	0.02
WS	2.12	0.06	0.38	0.15	0.43	0.13	0.03	0.05	7.97	1.74	0.08	0.37	0.16
Wr	0.83	0.36	0.29	0.58	0.40	0.43	0.18	0.27	10.67	0.32	0.14	0.26	0.26
**Sample**	**Se**	**Sn**	**Sr**	**Te**	**Th**	**Ti**	**Tl**	**U**	**V**	**W**	**Y**	**Zn**	**La**
RG	0.25	0.09	0.02	0.03	0	0	0	0	0	0.01	0	0.32	0
RH	0.22	0.87	0.08	0.33	0.01	0.01	0.01	0.01	0.01	0.05	0.01	0.73	0.01
RS	0.38	0.30	0.42	0.12	0.05	0.06	0.08	0.16	0.09	0.07	0.07	0.70	0.07
Rr	1.06	0.64	0.69	0.38	0.12	0.30	0.36	0.55	0.42	0.29	0.19	0.87	0.18
WG	0.47	0.12	0.05	0.00	0.01	0.01	0.01	0.01	0.01	0.01	0.01	0.63	0.01
WS	0.39	1.79	0.41	0.33	0.02	0.03	0.04	0.04	0.03	0.03	0.03	0.65	0.03
Wr	0.65	0.56	0.59	0.17	0.04	0.18	0.21	0.21	0.27	0.15	0.07	0.55	0.07
**Sample**	**Ce**	**Pr**	**Nd**	**Sm**	**Eu**	**Gd**	**Tb**	**Dy**	**Ho**	**Er**	**Tm**	**Yb**	**Lu**
RG	0.002	0.002	0.002	0.002	0.003	0.002	0.002	0.002	0.003	0.002	0.003	0.003	0.003
RH	0.01	0.01	0.01	0.01	0.01	0.01	0.01	0.01	0.01	0.01	0.01	0.01	0.01
RS	0.06	0.07	0.07	0.07	0.07	0.07	0.07	0.07	0.07	0.07	0.07	0.06	0.06
Rr	0.17	0.20	0.21	0.22	0.22	0.20	0.21	0.22	0.22	0.22	0.21	0.20	0.19
WG	0.01	0.01	0.01	0.01	0.01	0.01	0.01	0.01	0.01	0.01	0.01	0.01	0.01
WS	0.03	0.03	0.03	0.03	0.04	0.03	0.03	0.03	0.03	0.03	0.03	0.03	0.03
Wr	0.11	0.10	0.10	0.10	0.11	0.09	0.10	0.10	0.10	0.09	0.09	0.09	0.08

RG, RH, RS, Rr, WG, WS, and Wr stand for rice grain, rice husk, rice straw, rice root, wheat grain, wheat straw, and wheat root, respectively.

Table S4. Translocation (Tr) factors of elements in rice and wheat cultivated in Ambagarh Chowki.

**Tr**	**Al**	**As**	**Ba**	**Be**	**Bi**	**Ca**	**Cd**	**Co**	**Cr**	**Cu**	**Fe**	**Ga**	**Ge**
RG/Rr	0.04	0.01	0.01	0.01	0.02	0.12	0.06	0.01	0.04	0.20	0.01	0.01	0.02

RH/Rr	0.14	0.18	0.09	0.04	0.14	0.17	0.15	0.05	1.08	3.53	0.09	0.06	0.24
RS/Rr	0.48	0.29	0.61	0.20	0.24	0.70	1.11	0.34	0.33	0.84	0.18	0.25	0.37
WG/Wr	0.14	0.44	0.09	0.04	0.09	0.13	0.19	0.03	0.08	0.23	0.04	0.05	0.06
WS/Wr	0.40	0.56	0.72	0.17	0.46	0.70	0.59	0.12	0.64	9.60	0.14	0.16	0.36
**Tr**	**K**	**Li**	**Mg**	**Mn**	**Mo**	**Na**	**Nb**	**Ni**	**P**	**Pb**	**Rb**	**Sb**	**Sc**
RG/Rr	0.26	0.14	0.03	0.13	0.03	0.02	0.02	0.01	0.01	0.05	0.02	0.37	0.23
RH/Rr	0.24	1.26	0.13	0.67	0.12	0.09	0.07	0.04	0.04	0.25	0.07	0.85	0.36
RS/Rr	0.37	0.46	0.69	0.33	0.40	0.33	0.25	0.49	0.27	0.36	0.36	0.80	0.66
WG/Wr	0.59	0.24	0.09	0.00	0.20	0.05	0.04	0.03	0.03	0.08	0.11	1.15	0.08
WS/Wr	0.57	4.31	0.72	0.00	0.58	0.18	0.21	0.18	0.12	0.25	0.37	1.20	0.32
**Tr**	**Se**	**Sn**	**Sr**	**Te**	**Th**	**Ti**	**Tl**	**U**	**V**	**W**	**Y**	**Zn**	**La**
RG/Rr	0.26	0.14	0.03	0.13	0.03	0.02	0.02	0.01	0.01	0.05	0.02	0.37	0.23
RH/Rr	0.24	1.26	0.13	0.67	0.12	0.09	0.07	0.04	0.04	0.25	0.07	0.85	0.36
RS/Rr	0.37	0.46	0.69	0.33	0.40	0.33	0.25	0.49	0.27	0.36	0.36	0.80	0.66
WG/Wr	0.59	0.24	0.09	0.00	0.20	0.05	0.04	0.03	0.03	0.08	0.11	1.15	0.08
WS/Wr	0.57	4.31	0.72	0.00	0.58	0.18	0.21	0.18	0.12	0.25	0.37	1.20	0.32
**Tr**	**Ce**	**Pr**	**Nd**	**Sm**	**Eu**	**Gd**	**Tb**	**Dy**	**Ho**	**Er**	**Tm**	**Yb**	**Lu**
RG/Rr	0.01	0.01	0.01	0.01	0.01	0.01	0.01	0.01	0.01	0.01	0.01	0.01	0.01
RH/Rr	0.07	0.06	0.06	0.06	0.07	0.06	0.06	0.06	0.06	0.06	0.06	0.06	0.07
RS/Rr	0.36	0.35	0.34	0.32	0.36	0.33	0.31	0.30	0.30	0.30	0.30	0.30	0.31
WG/Wr	0.08	0.08	0.07	0.07	0.07	0.08	0.07	0.07	0.07	0.07	0.07	0.07	0.08
WS/Wr	0.27	0.28	0.26	0.28	0.34	0.28	0.27	0.27	0.27	0.27	0.26	0.28	0.31

RG, RH, RS, Rr, WG, WS, and Wr stand for rice grain, rice husk, rice straw, rice root, wheat grain, wheat straw, and wheat root, respectively.

Table S5. Health hazard parameters of toxic elements for cereal samples.

Element	Grain	ATD	CDI	HQ	CR
As	RG	2.41±0.04	0.043±0.036	133.8±113.2	0.0602±0.0509
	MG	5.98±0.10	0.105±0.007	279.4±8.1	0.1258±0.0037
	WG	5.04±0.08	0.089±0.002	332.3±23.4	0.1566±0.011
Cr	RG	2.67±0.04	0.0473±0.037	14.8±11.6	0.0223±0.0174
	MG	1.24±0.02	0.022±0.009	4.1±0.9	0.0062±0.0014
	WG	0.74±0.01	0.013±0.003	6.9±2.9	0.0103±0.0043
Ni	RG	0.96±0.02	0.017±0.012	0.80±0.56	0.0272±0.0190
	MG	0.43±0.01	0.007±0.002	0.27±0.01	0.0091±0.0004
	WG	0.32±0.01	0.006±0.0005	0.36±0.12	0.0123±0.0040
Pb	RG	1.14±0.02	0.020±0.009	5.42±2.45	0.0072±0.0033
	MG	1.27±0.02	0.022±0.019	0.84±0.20	0.0011±0.0003
	WG	0.17±0	0.003±0.0008	6.04±5.16	0.0080±0.0069
Cd	RG	0.009±0	0.0002±0.002	0.159±0.143	$1.6±1.4 \times 10^{-6}$
	MG	0.030±0.001	0.0004±0.00005	0.044±0.016	$4.4±1.6 \times 10^{-7}$
	WG	0.003±0	0.00005±0.00001	0.417±0.049	$4.2±0.5 \times 10^{-6}$

ATD, CDI, CR, and HQ stand for average total dose, chronic daily intake, cancer risk, and hazard quotient, respectively. RG, WG, and MG stand for rice grain, wheat grain, and maize grain, respectively.

Table S6. Factor loadings indicating the contribution of elements measured in grains to each factor in the factor analysis-based source apportionment.

Element	F1	F2	F3
Al	0.96		

As	0.92		
Ba	0.91	0.33	
Be	0.96		
Bi	0.95		
Ca	0.91	0.34	
Cd	0.61		
Co	0.96		
Cr	0.61	0.46	
Cu		0.94	
Fe	0.99		
K		0.79	
Li	0.98		
Mg			0.80
Mn	0.75		
Mo			0.79
Na	0.60		
Ni	0.97		
P			0.58
Pb		0.94	
Rb	0.56		0.39
Sr	0.89	0.40	
Th	0.92		
Ti	0.98		
U	0.97		
V	0.98		

W	0.98		
Y	0.93		
Zn	0.37	0.60	
Eigen value	17.85	4.05	2.10
% of variance	61.55	13.98	7.25
Cumulative %	61.55	75.53	82.78
